# Chemical Structure and Anticoagulant Property of a Novel Sulfated Polysaccharide from the Green Alga *Cladophora oligoclada*

**DOI:** 10.3390/md19100554

**Published:** 2021-09-29

**Authors:** Meijia He, Yajing Yang, Zhuling Shao, Junyan Zhang, Changning Feng, Lei Wang, Wenjun Mao

**Affiliations:** 1 Key Laboratory of Marine Drugs of Ministry of Education, Shandong Provincial Key Laboratory of Glycoscience and Glycotechnology, School of Medicine and Pharmacy, Ocean University of China, Qingdao 266003, China; hmj@stu.ouc.edu.cn (M.H.); yangyajing@stu.ouc.edu.cn (Y.Y.); shaozhuling@stu.ouc.edu.cn (Z.S.); 21190811060@stu.ouc.edu.cn (J.Z.); 21190831023@stu.ouc.edu.cn (C.F.); 21190831043@stu.ouc.edu.cn (L.W.); 2 Laboratory for Marine Drugs and Bioproducts, Pilot National Laboratory for Marine Science and Technology (Qingdao), Qingdao 266237, China

**Keywords:** alga, sulfated polysaccharide, structure, anticoagulant property, action mechanism

## Abstract

Marine macroalgae are efficient producers of sulfated polysaccharides. The algal sulfated polysaccharides possess diverse bioactivities and peculiar chemical structures, and represent a great potential source to be explored. In the present study, a heparinoid-active sulfated polysaccharide was isolated from the green alga *Cladophora oligoclada.* Results of chemical and spectroscopic analyses indicated that the sulfated polysaccharide was composed of →6)-*β*-d-Gal*p*-(1→, *β*-d-Gal*p*-(1→, →6)-*α*-d-Glc*p*-(1→ and →3)-*β*-d-Gal*p*-(1→ units with sulfate esters at C-2/C-4 of →6)-*β*-d-Gal*p*-(1→, C-6 of →3)-*β*-d-Gal*p*-(1→ and C-3 of →6)-*α*-d-Glc*p*-(1→ units. The branches consisting of *β*-d-Gal*p*-(1→ and →6)-*β*-d-Gal*p*-(1→ units were located in C-3 of →6)-*β*-d-Gal*p*-(1→ units. The sulfated polysaccharide exhibited potent anticoagulant activity in vitro and in vivo as evaluated by activated partial thromboplastin time (APTT), thrombin time, and the fibrinogen level. For the APTT, the signal for clotting time was more than 200 s at 100 μg/mL in vitro and at 15 mg/kg in vivo. The obvious thrombolytic activity of the sulfated polysaccharide in vitro was also found. The mechanism analysis of anticoagulant action demonstrated that the sulfated polysaccharide significantly inhibited the activities of all intrinsic coagulation factors, which were less than 1.0% at 50 μg/mL, but selectively inhibited common coagulation factors. Furthermore, the sulfated polysaccharide strongly stimulated the inhibition of thrombin by potentiating antithrombin-III (AT-III) or heparin cofactor-II, and it also largely promoted the inhibition of factor Xa mediated by AT-III. These results revealed that the sulfated polysaccharide from *C. oligoclada* had potential to become an anticoagulant agent for prevention and therapy of thrombotic diseases.

## 1. Introduction

Arterial or venous blood clots can result in acute coronary syndrome or venous thromboembolisms, which are the third leading causes of cardiovascular-event related deaths [1,2]. Anticoagulant drugs represented by heparin have been widely used in the therapy and prophylaxis of thrombosis. However, heparin exhibits some side effects, such as thrombocytopenia, hemorrhagic complications, and congenital or acquired antithrombin deficiency invalidation [3]. Moreover, commercial manufacturing of heparin relies on pig intestinal or bovine lung tissues as the raw material, whereas these tissues presumably have a high burden of foreign parasites and may limit the use of heparin [4]. Thus, it is crucial to search for an alternative source of an anticoagulant agent.

Sulfated polysaccharides biosynthesized by marine algae are great potential sources of naturally occurring anticoagulant agents [5]. Sulfated galactans and sulfated fucans from red and brown algae are currently the most studied non-mammalian sulfated polysaccharides in glycomics [6,7,8,9]. The two types of algal sulfated polysaccharides comprise of new carbohydrate-based therapeutics for thrombotic diseases. Nevertheless, the extent and mechanism of action vary extremely due to their chemical structures and the degree of sulfation. Ulvan derived from green algae is also an important class of sulfated polysaccharides and exhibits potential anticoagulant activity [5]. Other heparinoid-active sulfated polysaccharides with different carbohydrate backbones from green algae have also been discovered [10,11,12]. The green algal sulfated polysaccharides are by far only partly explored due to the great diversity, and the metabolite complexity of green algae offer a unique and exclusive source of anticoagulant polysaccharides [13,14].

*Cladophora* is a group of macroscopic green algae with over 183 species [15]. Up to now, few works have correlated the structure and biological activity of sulfated polysaccharides from the genus *Cladophora.* The sulfated xylogalactoarabinan from *C. falklandica,* which consisted of a backbone of 4-linked -l-arabinopyranose units, showed anticoagulant activity, and it modified the kinetics of fibrin formation, reduced the time of permanence of the clot in the blood vessel, and induced a lesser thrombogenic state [16]. In addition, the nitric oxide releasing capacity of the polysaccharide extracted from *C. glomerata* Kützing was investigated, and the sulfate groups were found to be a key factor in regulating the immunomodulatory activity [17]. In the present research, a heparinoid-active sulfated polysaccharide was isolated from *C. oligoclada,* which is widely distributed through the world’s seas, and the characteristics of its structure and anticoagulant property were studied. The results reveal that the sulfated polysaccharide has a high potential as a drug or a food supplement for prevention and therapy of thrombotic diseases.

## 2. Results and Discussion

### 2.1. Structural Characteristics of the Sulfated Polysaccharide

**Figure 1:**
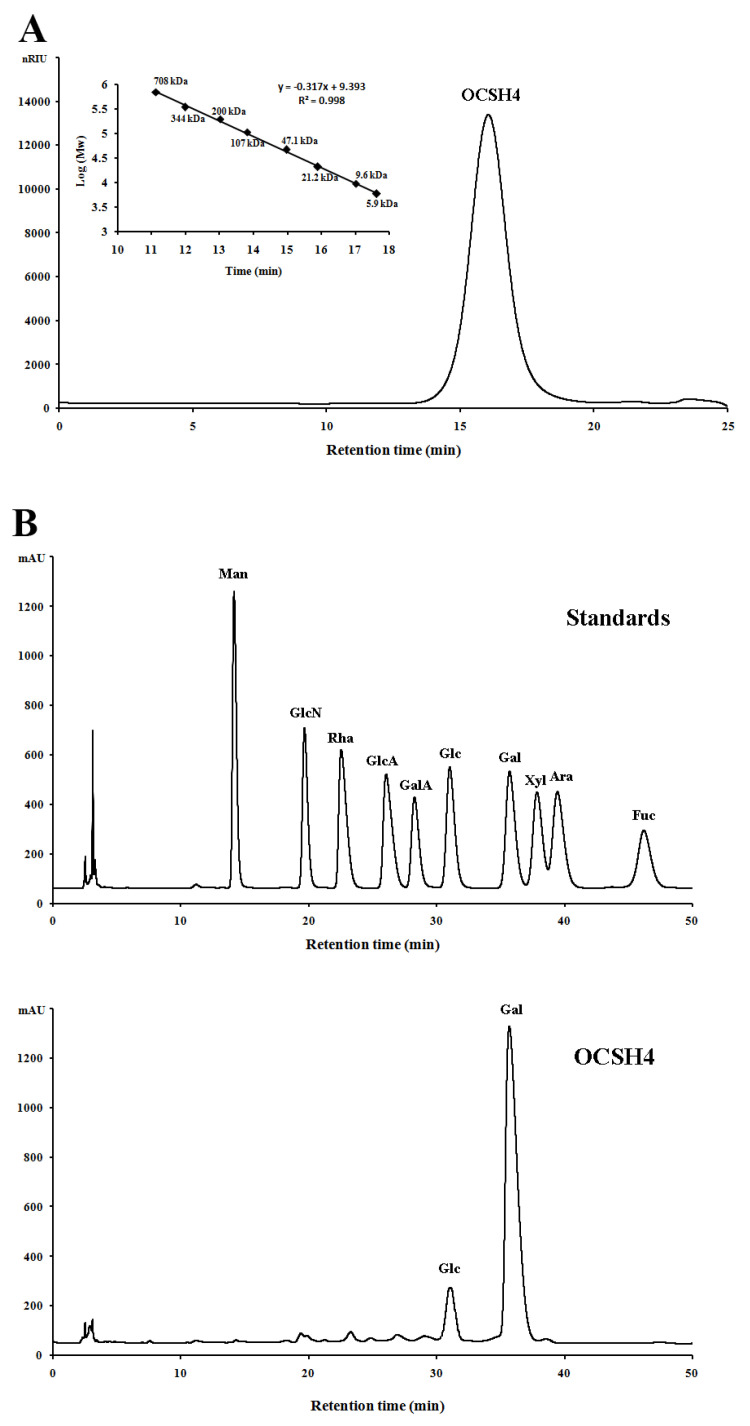

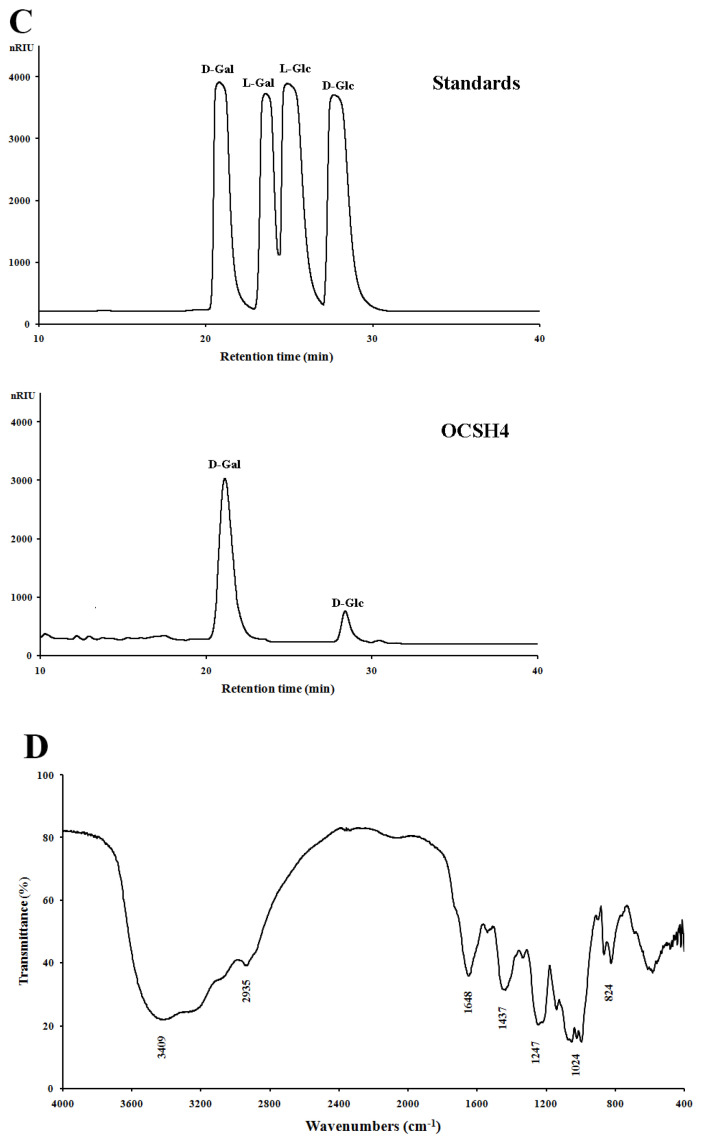
HPGPC and HPLC chromatograms and IR spectrum of OCSH4. (**A**) HPGPC chromatogram of OCSH4 on a Shodex OHpak SB-804 HQ column and the standard curve of molecular weight. (**B**) HPLC chromatogram for the monosaccharide composition analysis of OCSH4 (Man: d-mannose, GlcN: d-glucosamine, Rha: l-rhamnose, GlcA: d-glucuronic acid, GalA: d-galacturonic acid, Glc: d-glucose, Gal: d-galactose, Xyl: d-xylose, Ara: l-arabinose, Fuc: l-fucose). (**C**) HPLC chromatogram for the sugar configuration determination of OCSH4; and (**D**) IR spectrum of OCSH4.

The sulfated polysaccharide from the green alga *C. oligoclada*, designated OCSH4, was obtained by extraction with water, and purified with ion exchange and gel filtration chromatography, giving a yield of 1.25% (*w*/*w*) of the dry alga. OCSH4 produced only a symmetrical peak in the high performance gel permeation chromatography (HPGPC) chromatogram, and corresponded to a molecular mass of 18.1 kDa (Figure 1A). Chemical assays showed that OCSH4 contained 38.9% sulfate ester without protein and uronic acid. A high performance liquid chromatography (HPLC) assay indicated that OCSH4 consisted of galactose (89.39%) and glucose (10.61%) (Figure 1B). Based on the results of the HPLC analysis, the galactose and glucose in OCSH4 were deduced to be in the d-configuration (Figure 1C). IR spectrum of OCSH4 exhibited bands corresponding to sulfate esters, 824 cm^−^^1^ (C–O–S stretching vibration), and 1247 cm^–^^1^ (S–O tensile vibration) (Figure 1D). The peaks appeared at 3409 and 2935 cm^–1^, and were the results of the existence of O–H and C–H groups. The bands at 1648, 1437, and 1024 cm^–1^ corresponded to the peaks of C=O, C–H, and C–O–H groups, respectively.

In order to gain more structural features of OCSH4, the desulfated product of OCSH4 was prepared. The bands at 1247 and 824 cm^–1^ disappeared in the IR spectrum of dsOCSH4 (Figure S1A), indicating that almost all sulfate ester was removed. HPGPC chromatogram of dsOCSH4 showed a single peak and no peaks appeared in the oligomer region (Figure S1B); thus, no glycosidic bond was destroyed in the process of desulfation. The molecular weight of dsOCSH4 was about 15.0 kDa. The total sugar content of dsOCSH4 was 98.2%, and it contained 0.5% sulfate ester. In comparison with the result of OCSH4, monosaccharide composition of dsOCSH4 almost had no change (Figure S1C).

Methylation analysis showed that OCSH4 was mainly composed of →6)-Gal*p*-(1→ and →3,6)-Gal*p*-(1→ residues, followed by Gal*p*-(1→, →4,6)-Gal*p*-(1→, →3,6)-Glc*p*-(1→ and →2,6)-Gal*p*-(1→ residues (Table 1). Compared with the methylation result of dsOCSH4, →6)-Gal*p*-(1→ residue increased, whereas the →3,6)-Gal*p*-(1→ residue decreased. The →3)-Gal*p*-(1→ and →6)-Glc*p*-(1→ residues appeared, while the →4,6)-Gal*p*-(1→, →3,6)-Glc*p*-(1→ and →2,6)-Gal*p*-(1→ residues disappeared. Thus, it was speculated that the sulfate groups mainly substituted at C-3 of the →6)-Glc*p*-(1→ residue, C-6 of the →3)-Gal*p*-(1→ residue and C-2/C-4 of the →6)-Gal*p*-(1→ residue. The presence of →3,6)-Gal*p*-(1→ residue in dsOCSH4 indicated that OCSH4 had side chains at C-3 of →6)-Gal*p*-(1→ residue.

**Figure 2:**
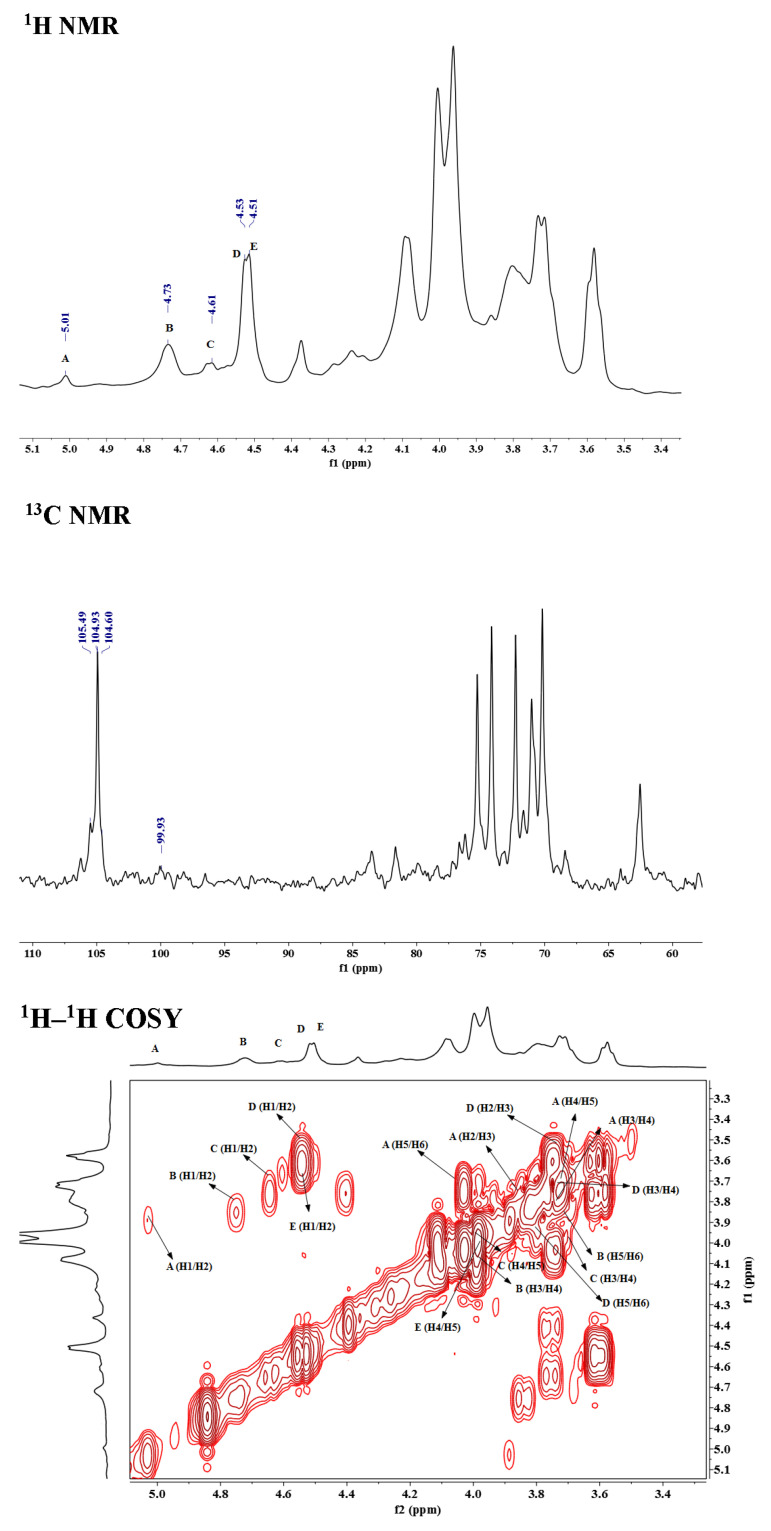

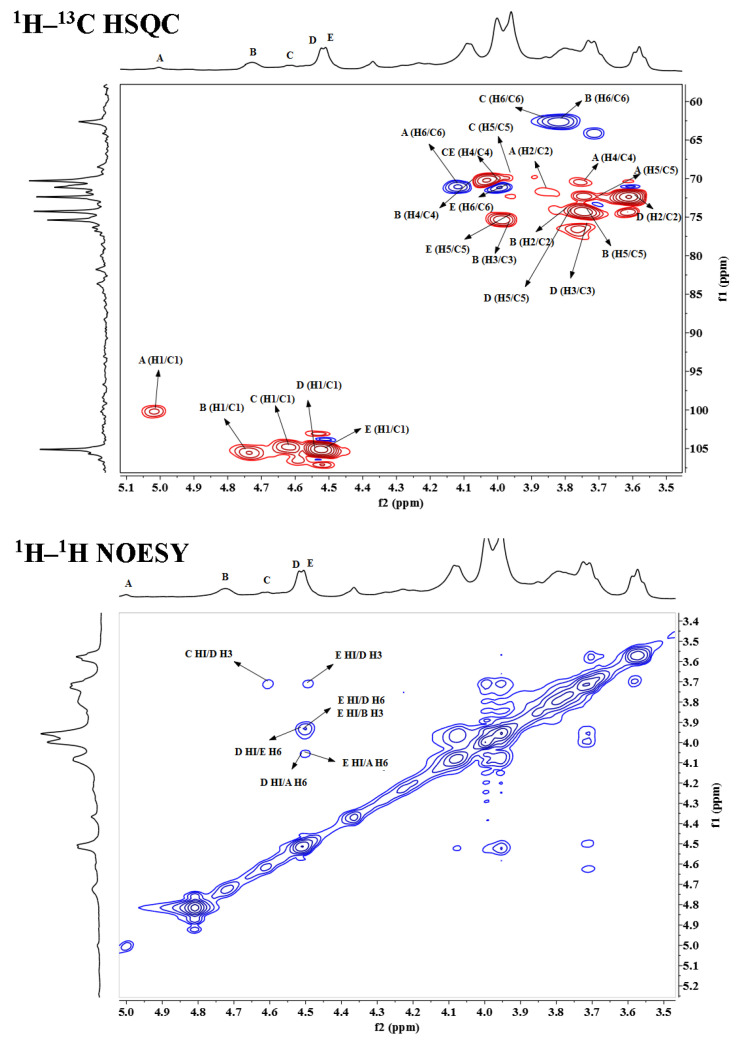
NMR spectra of dsOCSH4. A-E: →6)-*α*-d-Glc*p*-(1→, →3)-*β*-d-Gal*p*-(1→, *β*-d-Gal*p*-(1→, →3,6)-*β*-d-Gal*p*-(1→ and →6)-*β*-d-Gal*p*-(1→. Glc*p*: glucopyranose, Gal*p*: galactopyranose.

To elucidate the structure of OCSH4, the NMR spectra of dsOCSH4 (Figure 2) was firstly analyzed. From the ^1^H NMR spectrum of dsOCSH4, five types of glycosidic linkages were speculated according to anomeric proton signals at 5.01, 4.73, 4.61, 4.53, and 4.51 ppm. The anomeric carbon signals appeared at 104.99, 104.42, and 104.09 ppm, and were ascribed to C-1 of *β*-d-galactopyranose units [18]. The signal at 99.43 ppm could be the C-1 of *α*-d-glucopyranose residue [19]. The ^1^H–^1^H COSY spectrum of dsOCSH4 demonstrated the proton correlations of the sugar residues. Accompanied with the ^1^H–^13^C HSQC spectrum and reference to previous reports [14,20,21], assignments of the signals of dsOCSH4 were completed (Table 2). The anomeric signals of the five linkages were presented as 5.01/99.43 ppm (A), 4.73/104.99 ppm (B), 4.61/104.09 ppm (C), 4.53/104.99 ppm (D) and 4.51/104.42 ppm (E). The residue A with the anomeric signals H-1/C-1 (5.01/99.43 ppm) was assigned to 6-linked *α*-d-glucopyranose because of the chemical shift of C-6 (69.59 ppm). The residue B was ascribed to →3)-*β*-d-Gal*p*-(1→ due to the downfield shifts of H-3/C-3 (3.96/75.63 ppm). The cross-peaks of H-1/C-1(4.61/104.09 ppm) and correlated signals from H-2/C-2 to H-6/C-6 in the HSQC spectrum indicated that the residue C was *β*-d-Gal*p*-(1→. The residue D was attributed to →3,6)-*β*-d-Gal*p*-(1→ in view of the downfield shifts of C-3 (76.06 ppm) and C-6 (69.68 ppm). The residue E was ascribed to →6)-*β*-d-Gal*p*-(1→ due to the downfield shifts of H-6/C-6 (3.96/69.68 ppm).

**Figure 3:**
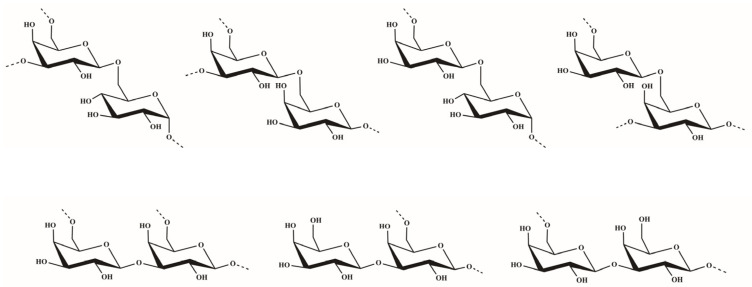
Structures of the major disaccharides in dsOCSH4.

The sequences of glycosyl residues were proposed by the ^1^H–^1^H NOESY spectrum. In the ^1^H–^1^H NOESY spectrum of dsOCSH4, the obvious anomeric proton signal of the residue D was correlated to the H-6 of the residues A and E, confirming the presence of →3,6)-*β*-d-Gal*p*-(1→6)-*α*-d-Glc*p*-(1→ and →3,6)-*β*-d-Gal*p*-(1→6)-*β*-d-Gal*p*-(1→. The correlations between H-1 (E)/H-6 (A/D) proved the fragments →6)-*β*-d-Gal*p*-(1→6)-*α*-d-Glc*p*-(1→ and →6)-*β*-d-Gal*p*-(1→3,6)-*β*-d-Gal*p*-(1→. The cross signals H-1 (E)/H-3 (D) showed that the H-1 of →6)-*β*-d-Gal*p*-(1→ residue was attached to the H-3 of →3,6)-*β*-d-Gal*p*-(1→ residue. As the same, the cross signals H-1 (C)/H-3 (D) indicated that the H-1 of *β*-d-Gal*p*-(1→ residue was attached to the H-3 of →3,6)-*β*-d-Gal*p*-(1→ residue. Furthermore, the two cross signals H-1 (E)/H-3 (D) and H-1 (C)/H-3 (D) confirmed that the side chains consisting of *β*-d-Gal*p*-(1→ and →6)-*β*-d-Gal*p*-(1→ units were located in C-3 of →6)-*β*-d-Gal*p*-(1→ units. In addition, the anomeric proton signal of the residue E was correlated to the H-3 of the residue B, confirming the presence of →6)-*β*-d-Gal*p*-(1→3)-*β*-d-Gal*p*-(1→. The structures of the major disaccharides in dsOCSH4 are listed in Figure 3.

The NMR spectra of OCSH4 (Figure S2) was further elucidated. The ^1^H NMR spectrum of OCSH4 showed seven anomeric proton signals at 5.09, 4.76, 4.62, 4.60, 4.59, 4.53, and 4.50 ppm. The related anomeric carbon signals in the ^13^C NMR spectrum of OCSH4 were 104.88, 104.30, 103.03, and 99.57 ppm. Among them, the signal at 99.57 ppm was assigned to *α*-linked glucopyranose unit, whereas the other signals were assigned to *β*-linked galactopyranose units. All chemical shifts of ^1^H and ^13^C are listed in Table 3. The intensive anomeric proton signal of A at 5.09 ppm indicated it was an *α*-linked unit, and its anomeric carbon signal was at 99.57 ppm. Compared with the signals of →6)-*α*-d-Glc*p*-(1→ and the related signals H-3/C-3 (4.48/79.55 ppm) of A, the residue A was attributed to →6)-*α*-d-Glc*p*(3SO_4_)-(1→. For the residues B, D, and E, the anomeric proton signals at 4.76, 4.60, and 4.59 ppm were correlated to the anomeric carbon signals at 103.03, 104.30, and 104.30 ppm, respectively. Compared with the chemical shifts of →6)-*β*-d-Gal*p*-(1→ residue, H-2/C-2 of the residue B changed to low field at 4.40/79.64 ppm indicated that the residue B was sulfated at C-2. Similarly, the low field shifts of H-4/C-4 (4.43/77.34 ppm) confirmed that the residue E was →6)-*β*-d-Gal*p*(4SO_4_)-(1→. Compared with the data of →3)-*β*-d-Gal*p*-(1→ residue, and the low field shift at C-6 (72.26 ppm) of D indicated that the residue D was →3)-*β*-d-Gal*p*(6SO_4_)-(1→. Furthermore, OCSH4 contained three same residues with dsOCSH4, as visualized in COSY and HSQC spectra, the residues C, F, and G were assigned to *β*-d-Gal*p*-(1→, →3,6)-*β*-d-Gal*p*-(1→ and →6)-*β*-d-Gal*p*-(1→, respectively.

The repeating linkages of sugar residues were speculated by ^1^H–^13^C HMBC and ^1^H–^1^H NOESY spectra. Based on the analysis of ^1^H–^13^C HMBC spectrum, the correlated signals among H-1 (D)/C-6 (A/B/E/F/G), H-1 (E)/C-6 (A/B/F/G), and H-1 (F)/C-6 (A/B/E/G) established thirteen kinds of the repeating sequences in OCSH4. The cross signals of H-1 (D)/H-6 (A/B/E/F/G), H-1 (E)/H-6 (A/B/F/G), and H-1 (F)/H-6 (A/B/E/G) also appeared in NOESY spectrum, which confirmed the presence of these fragments in OCSH4. Moreover, the anomeric proton signal of G was correlated to the H-6 of A, B, E, and F in ^1^H–^1^H NOESY spectrum; thus, the fragments were confirmed as →6)-*β*-d-Gal*p*-(1→6)-*α*-d-Glc*p*(3SO_4_)-(1→, →6)-*β*-d-Gal*p*-(1→6)-*β*-d-Gal*p*(2SO_4_)-(1→, →6)-*β*-d-Gal*p*-(1→6)-*β*-d-Gal*p*(4SO_4_)-(1→ and →6)-*β*-d-Gal*p*-(1→3,6)-*β*-d-Gal*p*-(1→.

Taken together, OCSH4 mainly consisted of →6)-*β*-d-Gal*p*-(1→, →3)-*β*-d-Gal*p*-(1→, *β*-d-Gal*p*-(1→ and →6)-*α*-d-Glc*p*-(1→ units, with sulfate groups at C-2/C-4 of the →6)-*β*-d-Gal*p*-(1→, C-3 of the →6)-*α*-d-Glc*p*-(1→ and C-6 of →3)-*β*-d-Gal*p*-(1→ units. The side chains consisting of *β*-d-Gal*p*-(1→ and →6)-*β*-d-Gal*p*-(1→ were at C-3 of →6)-Gal*p*-(1→ residue.

So far, the structure characteristics of polysaccharides from the genus *Cladophora* were seldom reported. The polysaccharide from *C. rupestris* had a highly branched structure with galactose, arabinose, and rhamnose residues in the main chain and xylose and galactose at the side chains [22]. Subsequently, a polysaccharide from *C. socialis* was reported, it mainly consisted of 4-linked arabinose and 3-linked galactose [23]. However, their structures were not fully characterized. The polysaccharide F1 extracted from *C. glomerata* Kützing proved to have the backbone of (1→4)-*α*-l-arabinopyranosyl residues with branches and sulfate esters mainly at the C-2 position [17]. The sulfated xylogalactoarabinans from *C. falklandica* were composed of a backbone of 4-linked *β*-l-arabinopyranose units partially sulfated, mainly at C-3 and at C-2. In addition, there was partial glycosylation, mostly on C-2 [16]. It was noted that these polysaccharides from the genus *Cladophora* were mainly arabinan-type sulfated polysaccharides. The structural characteristics of water-soluble polysaccharide OCSH4 from *C. oligoclada* were distinguished from those of the sulfated polysaccharides previously obtained from the genus *Cladophora*. OCSH4 was a glucose-containing sulfated galactan. The differences of structure in the sulfated polysaccharides could be derived from the species and the ecophysiological growth conditions. In addition, the extraction method of the polysaccharides might have an impact on the structure feature and biological activity of the polysaccharides [24]. The generalization degree of the structural characteristics in the genus *Cladophora* is not known yet, and requires further exploration.

### 2.2. Anticoagulant Activity In Vitro of OCSH4

**Figure 4:**
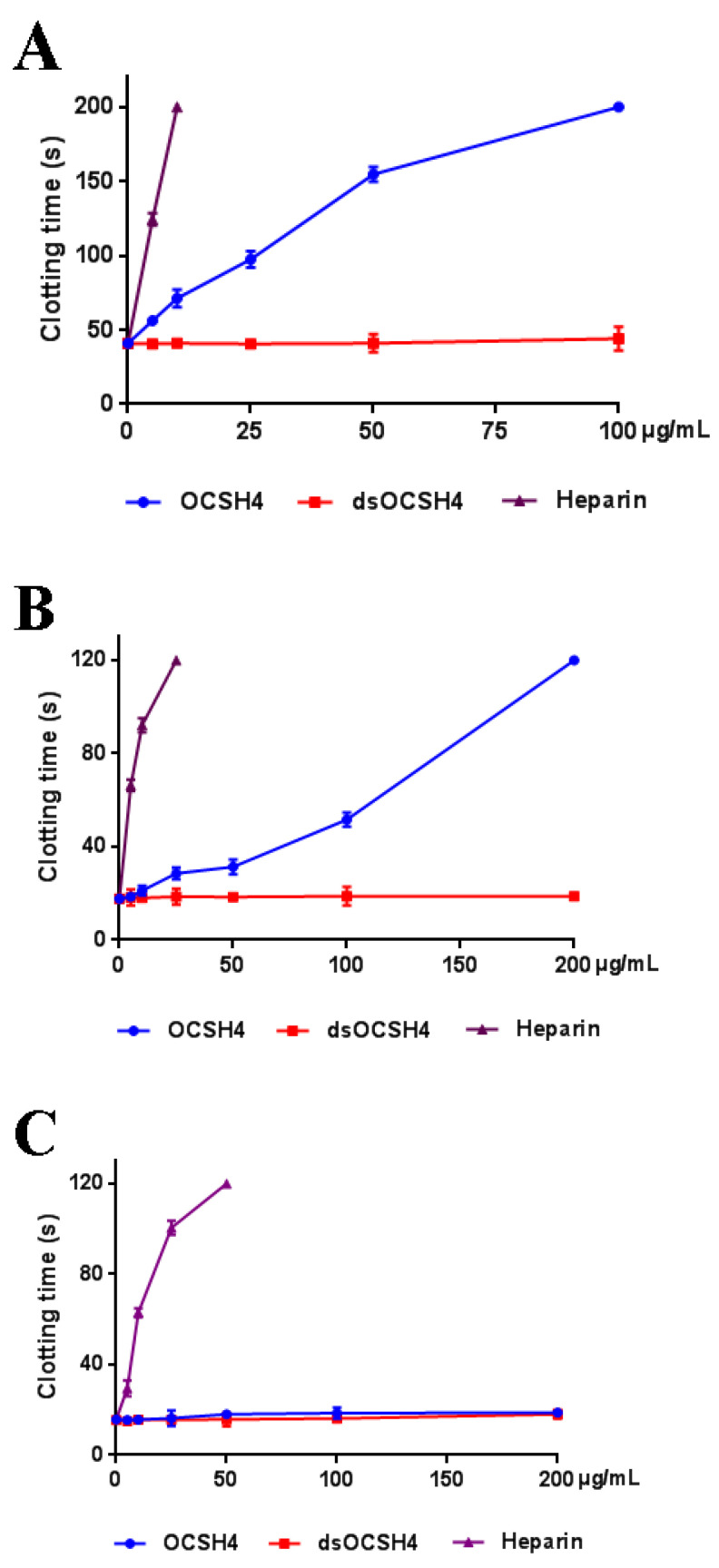

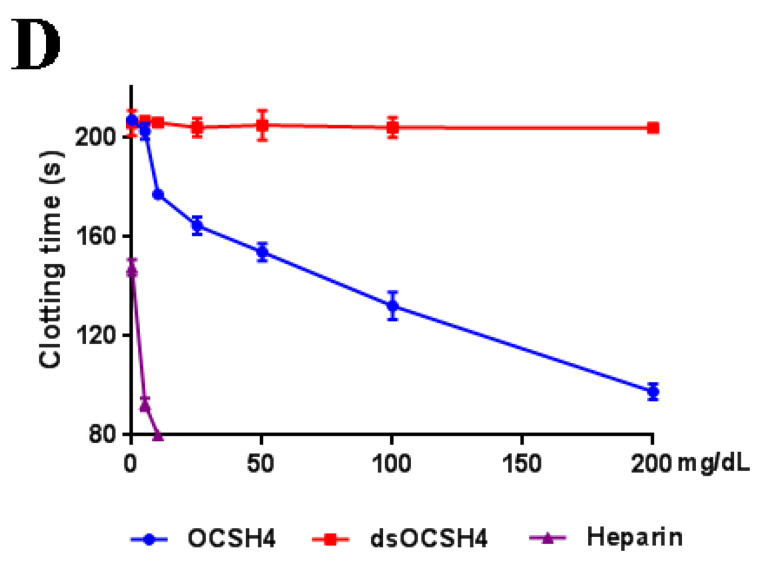
Anticoagulant activities of OCSH4 and dsOCSH4 in vitro. (**A**) APTT; (**B**) TT; (**C**) PT; and (**D**) FIB. Values were mean ± SD (*n* = 3).

Anticoagulant activity in vitro of OCSH4 was assessed by measuring the activated partial thromboplastin time (APTT), thrombin time (TT), prothrombin time (PT), and fibrinogen (FIB) (Figure 4). APTT was significantly prolonged by OCSH4 with increasing concentration of polysaccharide, and clotting time was more than 200 s at 100 µg/mL. TT activity (and effectively) increased in a concentration dependent manner, and the clotting time was more than 120 s at 200 µg/mL. Moreover, the APTT increase of OCSH4 was higher than that observed in TT. TT measures the common coagulation pathway, while APTT for the intrinsic coagulation pathway. No clotting inhibition was observed in PT test at the concentration assayed. PT is a sensitive screening test for the extrinsic coagulation pathway. The negligible effect of the obtained sulfated polysaccharide on PT was similar to other marine sulfated polysaccharides fucoidan obtained from brown algae and carrageenan from red algae. Hence, the monomeric composition does not have a significant effect in anti-coagulant activity [6,25]. It was observed that the APTT and TT activities by treatment with heparin rapidly increased, the coagulation time of APTT at 10 μg/mL exceeded 200 s, and that of TT exceeded 120 s at 25 μg/mL. The result suggested that OCSH4 might have a lower bleeding risk than heparin at the same therapeutic dose and could be a promising antithrombotic agent. In addition, with the increasing concentration of polysaccharide, the decrease of the FIB level induced by OCSH4 was observed. Fibrinogen is a key factor in coagulation. High fibrinogen levels can prevent platelet aggregation and, thus, prevent the formation of hypercoagulable states and thrombosis.

No anticoagulant activity of dsOCSH4 in vitro was observed at the concentrations assayed. The phenomenon has also been found for other types of sulfated polysaccharides. The desulfated product of sulfated arabinan from *Codium* *vermilara*, and less sulfated arabinan obtained from the same alga had less or no activity [26]. Qin et al. [14] reported that the sulfated arabinogalactan from *Chaetomorpha linum* showed potent anticoagulant activity in vitro, but its desulfated polysaccharide showed no activity. The sulfated rhamnan from the green alga *Monostroma* *nitidum* yielded a six-fold higher anticoagulant activity than heparin, but its desulfated product containing 8% of sulfate esters had no anticoagulant activity [10]. Thus, the anticoagulant activity of polysaccharide is closely related to the level of sulfate esters. However, the structural basis of the anticoagulant property is complex because it involves naturally heterogeneous polysaccharides, and further work is required.

### 2.3. Anticoagulant Activity In Vivo of OCSH4

Anticoagulant activity of OCSH4 in vivo was also investigated by APTT, PT, and TT assays (Table 4); no clotting inhibition was observed in the PT test at the concentrations assayed. On the contrary, APTT and TT were statistically prolonged by OCSH4 in a concentration-dependent manner. For APTT activity, the clotting time was more than 200 s at 15 mg/kg. Moreover, the prolongation effects of OCSH4 at 7.5 and 15 mg/kg on the APTT activity were significantly higher than that of heparin in the concentration assayed. The prolongation effect of OCSH4 at 7.5 mg/kg on the TT activity was similar to that of heparin in the concentration used in the experiment. The data suggested that OCSH4 possessed a strong anticoagulant activity in vivo.

The carrageenan from red algae and ulvan from green algae showed similar anticoagulant activity with OCSH4, which were effective in extending the APTT and TT, but poor in modulating PT [6,27]. OCSH4 had a higher anticoagulant activity than the sulfated polysaccharides from *Gentiana scabra* Bunge roots and the arabinogalactan-glycoconjugate from *Genipa americana* leaves [28,29], although all of them were galactan-type sulfated polysaccharides. In addition, it was noted that the ability of OCSH4 to stimulate prolongation of APTT or TT differed from that of the sulfated xylogalactoarabinan from *C. falklandica* [16]. The prolongation effect of the sulfated xylogalactoarabinan on the APTT activity was weaker than that on the TT activity, whereas the APTT increase of OCSH4 was higher than that observed in TT. The differences of anticoagulant property between them may be attributed to the structural variation. An in-depth investigation on the anticoagulant activity of sulfated polysaccharides with different structures will indubitably play an important role in the understanding of anticoagulant property.

### 2.4. Inhibition of Coagulation Factors II, V, X, VIII, IX, XI, and XII by OCSH4

The influences of OCSH4 on the coagulation factors of the intrinsic pathway (XII, XI, IX, and VIII) and the common pathway (II, V, and X) were evaluated (Table 5). The coagulation factors of the intrinsic pathway were effectively inhibited by OCSH4 in a dose-dependent manner, and their activities were less than 1.0% at 50 μg/mL. At a high concentration, the inhibition abilities of OCSH4 on coagulation factors XII, XI, IX, and VIII were similar to those of heparin. It was noted that the inhibitory effects of OCSH4 on common pathway factors II and V were weaker than those on the factors of the intrinsic pathway. In addition, OCSH4 did not have a significant effect on the factor X. The data showed that OCSH4 inhibited all the intrinsic coagulation factors, but selectively inhibited the common coagulation factors. Until now, few works have correlated the effects of green algal sulfated polysaccharides on coagulation factors. Qin et al. reported that the sulfated arabinogalactan from the green alga *Chaetomorpha linum* largely inhibited the activities of the coagulation factors XII, XI, IX, and VIII, but it had no inhibition effect on coagulation factor V [14]. The inhibition of coagulation factors XII, XI, IX, and VIII may be effective for the health promotion and treatment of cardiovascular disease [30]. The present data revealed that OCSH4 deserves to be studied as a potential anticoagulant.

### 2.5. Inhibition of Thrombin /Factor Xa by Heparin Cofactor-II (HC-II) or Antithrombin-III (AT-III) in the Presence of OCSH4

**Figure 5:**
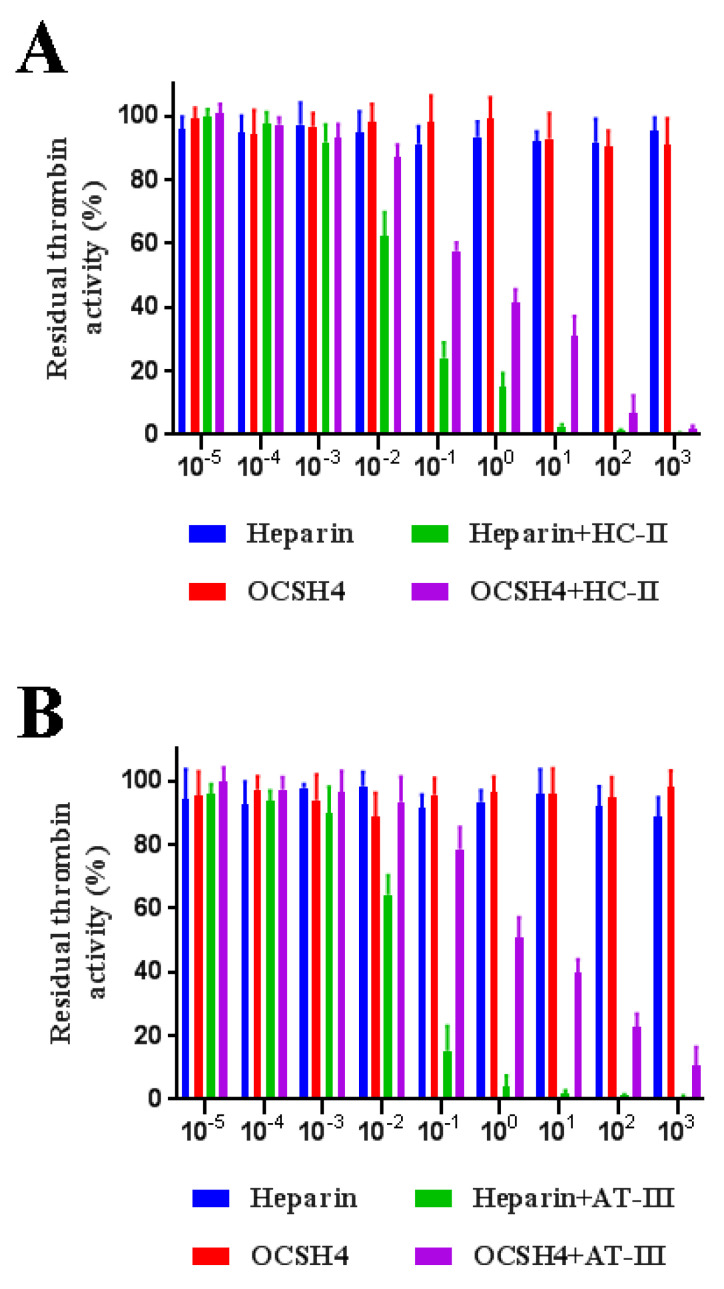

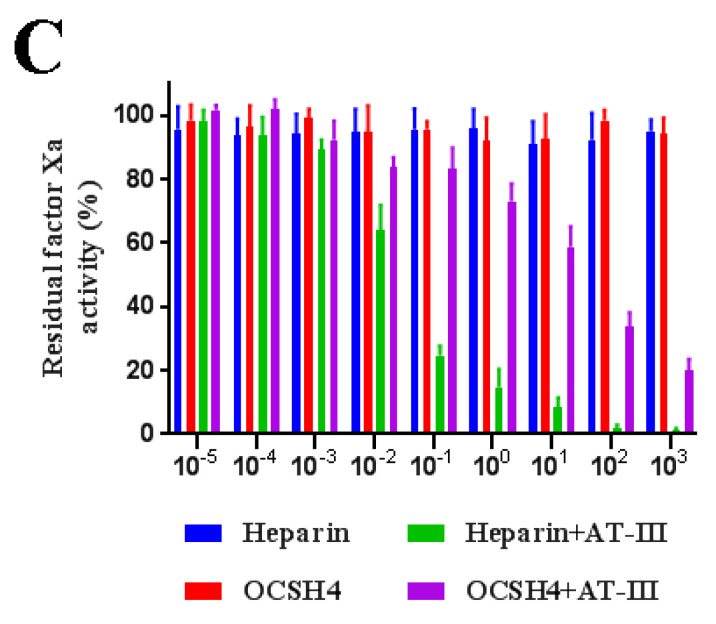
Inhibition of OCSH4 on HC-II/AT-III-dependent thrombin or factor Xa activity. Values were mean ± SD (*n* = 3). (**A**) Thrombin activity with or without of HC-II; (**B**) thrombin activity with or without AT-III; and (**C**) factor Xa activity with or without AT-III.

As shown in Figure 4, OCSH4 did not have a significant inhibition of thrombin in the absence of HC-II or AT-III. However, OCSH4 possessed a strong inhibition effect on thrombin through a HC-II-dependent pathway (Figure 5A). The distinct inhibition of the AT-III-dependent thrombin by OCSH4 was also observed (Figure 5B). It was noted that the factor Xa was not directly inhibited by OCSH4 in the absence of AT-III. OCSH4 had an obvious inhibition of factor Xa in the presence of AT-III, but its ability was not as strong as that of heparin (Figure 5C). Additionally, OCSH4 did not have an evident inhibition of factor Xa in the presence of HC-II (data not shown). AT-III inhibits all intrinsic pathway coagulation enzymes, whereas HC-II is a serine protease inhibitor and selectively inhibits thrombin [31]. These data revealed that OCSH4 could strongly stimulate the inhibition of thrombin mediated by HC-II or AT-III, and it effectively promoted the inhibition of factor Xa by AT-III.

Few reports on the inhibitory effects of sulfated polysaccharides from the genus *Cladophora* on thrombin and factor Xa have been presented. Arata et al. [16] found that the sulfated xylogalactoarabinans from the green alga *C. falklandica* exerted its anticoagulant activity through direct inhibition on thrombin activity. In addition, the anticoagulant activity of the sulfated polysaccharide from the green alga *Penicillus capitatus,* which was mainly constituted by (1→3)-*β*-d-galactose and (1→3,6)-*β*-d-galactose, was attributed to direct thrombin interaction [32]. The inhibition of thrombin by OCSH4 was different from that of the sulfated polysaccharide from *Penicillus capitatus*, although they mainly consisted of galactose. Comparison of the inhibition of the polysaccharides on thrombin activity indicated that complex relationships existed between the structure and anticoagulant property of the polysaccharides. The stereo-specificity of the structure of sulfated polysaccharides is an important factor for its interaction with coagulation inhibitors and their target proteases [33]. Different polysaccharides can form specific complexes with plasma inhibitors and target proteases. An in-depth study on the relationship between the structure and mechanism of anticoagulant activity of *Cladophora* sulfated polysaccharides is required.

### 2.6. Thrombolytic Activity In Vitro of OCSH4

**Figure 6:**
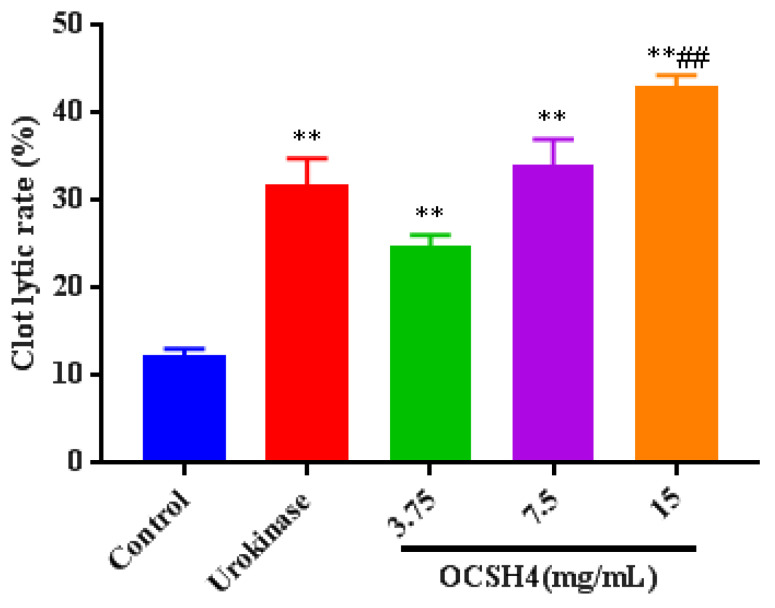
Thrombolytic activity in vitro of OCSH4. Values were mean ± SD (*n* = 10). Significance: ** *p* < 0.01 vs. the control group, ^##^ *p* < 0.01 vs. the model group.

Thrombolytic activity in vitro of OCSH4 was assessed by using urokinase as the positive control (Figure 6). The clot lytic rate of OCSH4 was markedly higher than that of control. Furthermore, the clot lytic rate of OCSH4 was in a concentration-dependent manner. The clot lytic rate of OCSH4 at 3.75 mg/mL was up to 24.4%, and the clot lytic rate at 7.5 mg/mL was remarkably higher than that of urokinase in the concentration assayed. These data illustrated that OCSH4 exhibited strong thrombolytic activity in vitro. Thus far, no report on the thrombolytic activity of sulfated polysaccharide from the genus *Cladophora* was found. It was noted that the clot lytic effect of OCSH4 was remarkably higher than those of sulfated rhamnan from *Monostroma* species [10,34] and sulfated arabinogalactan from *Chaetomorpha linum* [14]. The difference of thrombolytic property might be closely related to the structural characteristics of the sulfated polysaccharides, such as monosaccharide composition, proportion, and distribution of the sulfation pattern and glycosidic linkage. The present result revealed that OCSH4 from *C. oligoclada* could be a potential source of an anticoagulant. Further work on the thrombolytic activity in vivo of OCSH4 is still in progress.

The anticoagulant activity of algal sulfated polysaccharides is widely studied. It is known that fucan-containing polysaccharides have high anticoagulant activities, which depend on various factors, including molecular weight and sulfate content. The anticoagulant property of fucoidan from brown alga *Fucus evanescens* was similar to that of OCSH4, they were potent thrombin inhibitors mediated by AT-III [35]. There are many similarities between the heparin-like anticoagulant activities of sulfated polysaccharides from green and brown algae. For example, both polysaccharides could significantly prolong APTT and TT in vitro and in vivo, and the anticoagulant activities are affected by sulfation degree, monosaccharide content, and molecular mass [36,37]. Sulfated polysaccharides occur in high amounts in marine green algae. Algal anticoagulant polysaccharides hold great promise as a potential instead source of heparin from animal sources for therapy. However, their therapeutic use in humans faces several challenges. It is crucial to obtain more comprehensive information on the anticoagulant activity of algal sulfated polysaccharides, including the inhibition of coagulation factors. Nevertheless, the sulfated polysaccharide OCSH4 inhibited all intrinsic coagulation factors (XII, XI, IX, and VIII), indicating that OCSH4 could inhibit all pathways of endogenous coagulation cascade, the effect of inhibition was VIII > XII > XI > IX, but selectively inhibited the common coagulation factors. The prolongation effect of OCSH4 on the APTT activity was stronger than that on the TT activity, which suggested that OCSH4 might have a lower risk of bleeding, and could be developed into a novel anticoagulant agent for prevention and therapy of thrombotic disease.

## 3. Materials and Methods

### 3.1. Materials

*C. oligoclada* was collected from the coastal area of Duodao Sea (Rizhao, China) in April 2017 and identified by Professor Xiangzhong Gong of Ocean University of China (Qingdao, Shandong, China). The raw algae was thoroughly washed with tap water, air-dried at 40 °C in an oven, milled, and kept at 25 °C in a dry environment. Sephacryl S-400/HR and Q Sepharose Fast Flow were from GE Healthcare Life Sciences (Piscataway, NJ, USA). APTT, TT, PT, and FIB kits were from MD Pacific (Tianjin, China). Heparin (196 U/mg) was from Sigma (St. Louis, MO, USA). Factor Xa and thrombin were from Boatman Biotech CO., LTD. (Shanghai, China). HC-II was from Hyphen Biomed (Neuville, France). AT-III was from Chromogenix (Milan, Italy). Chromogenic substrates S-2765 and S-2238 were from Asnail (Beijing, China). Standard human plasma and a series of deficient human plasma (lacking factor XII, XI, IX, VIII, II, V, or X) were from Siemens (Munich, Germany).

### 3.2. Animals

Male Sprague–Dawley rats (180–220 g body weight) were housed at 23 ± 2 °C under a 12 h light/dark cycle with free access to food and water. All experiments involving animals were permitted by the Animal Care and Use Committee of Ocean University of China (OUC-YY-202001001).

### 3.3. Isolation of the Sulfated Polysaccharide

The alga powder (100 g) was extracted using 40-fold volume of H_2_O at room temperature for 4 h, and gave a crude polysaccharide (16.69 g). Then, the crude polysaccharide was separated by anion-exchange chromatography using a Q Sepharose Fast Flow column (45 mm × 65 cm, GE Healthcare Life Sciences, Piscataway, NJ, USA) and eluted with a step-wise gradient of 0–3 mol/L NaCl [34]. Total sugar content of each fraction was tested by the phenol–sulfuric acid method [38]; four main fractions were obtained under different elution conditions. The fraction eluted with 1.5 mol/L NaCl, which was the most abundant fraction, was pooled, dialyzed, and purified on a Sephacryl S-400/HR column (26 mm × 120 cm, GE Healthcare Life Sciences, Piscataway, NJ, USA) with 0.2 mol/L NH_4_HCO_3_ as eluent. Main fractions were pooled, concentrated, lyophilized, and named OCSH4 (1.25 g).

### 3.4. Component Analysis of OCSH4

Total sugar content was determined by the phenol–sulfuric acid method [38]. Sulfate content was measured according to the method of Therho and Hartiala [39]. Uronic acid content was estimated by the carbazole–sulfuric acid method [40]. Protein content was assayed as described by Bradford [41]. The molecular weight and purity were determined by HPGPC on a Shodex OHpak SB-804 HQ column (8.0 × 300 mm, Showa Denko K.K., Tokyo, Japan) [29]. The molecular weight was estimated by reference to a calibration curve made by pullulan standards (*Mw*: 5.9, 9.6, 21.1, 47.1, 107, 200, 344, and 708 kDa, Showa Denko K. K., Tokyo, Japan). Monosaccharide composition was measured by reversed-phase HPLC [14] using the Eclipse XDB-C18 column (4.6 × 250 mm, Agilent Technologies, Santa Clara, CA, USA). The chromatogram was performed on an Agilent 1260 Infinity HPLC instrument fitted with Agilent XDB-UV detector (254 nm). Identification and quantification of sugar were done by comparison with reference sugars. Sugar configuration was determined as described by Tanaka et al. [42]. Briefly, polysaccharide sample was degraded with 2 mol/L trifluoroacetic acid at 105 °C for 6 h. Then, the hydrolysate was heated with l-cysteine methyl ester in pyridine at 60 °C for 1 h. The o-tolyl isothiocyanate solution was added to the mixture, and was further heated at 60 °C for 1 h. The reaction mixture was analyzed on an Agilent 1260 Infinity HPLC instrument using an Eclipse XDB-C_18_ column (4.6 × 250 mm) and an Agilent XDB-UV detector (250 nm). Sugar configuration was identified by comparison with retention time of the derivatives of reference sugars. Desulfation of OCSH4 was carried out according to Miller and Blunt [43], the product was named as dsOCSH4.

### 3.5. Methylation Analyses of OCSH4 and dsOCSH4

The analysis was performed according to the method by Harris et al. [44]. Polysaccharide was dissolved in dimethyl sulfoxide, and methylated using NaH and methyl iodide. After hydrolysis with 2 mol/L trifluoroacetic acid at 105 °C for 6 h, the methylated sugar residues were converted to partially methylated alditol acetates by reduction with NaBH_4_, followed by acetylation with acetic anhydride. The product was assayed on a TRACE 1300-ISO GC–MS spectrometer (Thermo Scientific, Waltham, MA, USA).

### 3.6. FTIR Spectroscopy

The polysaccharide (2.0 mg) was ground with KBr, pressed into a 1 mm transparent sheet, and then determined on a Nicolet Nexus 470 spectrometer (Thermo Fisher Scientific, Waltham, MA, USA) with a scan range of 400–4000 cm^−1^.

### 3.7. NMR Spectroscopy

One-dimensional (1D) and 2D NMR spectra were performed on an Agilent DD2 500 MHz NMR spectrometer (Agilent Technologies Co. Ltd., Palo Alto, CA, USA) at 25 °C [45]. Polysaccharide (60 mg) was exchanged in 99.9% D_2_O three times. Deuterated acetone was used as internal standard at 2.225 ppm for ^1^H and 31.07 ppm for ^13^C.

### 3.8. Anticoagulant Activity In Vitro

Anticoagulant activity in vitro was performed based on APTT, TT, PT, and FIB assays [46,47]. Polysaccharide was dissolved in 0.9% NaCl at different concentrations (0, 5, 10, 25, 50, 100, and 200 µg/mL). APTT assay was carried out as follows: citrated human plasma (90 µL) was mixed with the polysaccharide (10 µL) and incubated at 37 °C for 1 min. Then, pre-warmed APTT assay reagent (100 µL) was added and incubated at 37 °C for 2 min. Then, 100 µL of pre-warmed 0.25 mol/L CaCl_2_ was added and clotting time was recorded. For the TT clotting assay, citrated human plasma (90 µL) was mixed with the polysaccharide (10 µL) and incubated at 37 °C for 1 min. Then, pre-warmed TT assay reagent (200 µL) was added and clotting time was recorded. For the PT clotting assay, citrated human plasma (90 µL) was mixed with polysaccharide (10 µL) and incubated at 37 °C for 1 min. Then, pre-warmed PT assay reagent (200 µL) was added and clotting time was recorded. For FIB content assay, citrated human plasma (90 μL) was mixed with polysaccharide (10 μL), then was diluted with imidazole buffer (900 μL). The diluted plasma sample (200 μL) was taken and incubated at 37 °C for 3 min, followed by the addition of the FIB assay reagent (100 μL) and clotting time was recorded. FIB content was estimated by reference to a calibration curve made by FIB constant value plasma. Heparin was used as a positive control. Saline solution (0.9% NaCl) was used as a control.

### 3.9. Anticoagulant Activity In Vivo

Anticoagulant activity in vivo was evaluated by APTT, TT, and PT assays using rat plasma. In briefly, Sprague–Dawley rats were randomly divided into six experimental groups (eight rats in each group). The rats were anesthetized with 30% ethyl carbamate and then injected with OCSH4 (1, 3.75, 7.5, 15 mg/kg) and heparin (1 mg/kg). Saline solution (0.9% NaCl) was used as a control. After 30 min, the rats were secured in the supine position, and the blood was taken out from the abdominal aorta. APTT, TT, and PT assays of the rat plasma were carried out using commercial kits according to above-mentioned methods.

### 3.10. Assay of Coagulation Factor II, V, X, VIII, IX, XI, or XII Activity

The assay was carried out according to the method by Takahashi and Hiraga [48]. Polysaccharide was dissolved in imidazole buffer (pH 7.3) at different concentrations (5, 10, 25, 50 μg/mL). For factor II, V, or X activity assay, re-dissolved standard human plasma (90 μL) was mixed with polysaccharide (10 μL); deficient human plasma (100 μL) was added and incubated at 37 °C for 1 min. Thereafter, pre-warmed PT reagent (100 μL) was added and clotting time was recorded. For factor XII, XI, IX, or VII activity assay, re-dissolved standard human plasma (90 μL) was mixed with polysaccharide (10 μL), deficient human plasma (100 μL) and APTT reagent (100 μL) were added, and incubated at 37 °C for 2 min. Then pre-warmed 0.025 mol/L CaCl_2_ (100 μL) was added and clotting time was recorded. The activity of coagulation factor was assessed by reference to calibration curve made by a series of dilutions of standard human plasma. Standard human plasma was diluted with imidazole buffer. The standard human plasma with a dilution ratio of 1:5 (plasma/imidazole buffer, *v/v)* was used as a control. The mean value of coagulation factor activity in the control was defined as 100%. Heparin was used as a positive control.

### 3.11. Inhibition of Thrombin/Factor Xa by HC-II or AT-III in the Presence of OCSH4

The assay was done according to the method by Colliec et al. [49]. Human thrombin or factor Xa and inhibitors (HC-II or AT-III) were incubated with or without the polysaccharide in 180 µL of 0.02 mol/L Tris–HCl (pH 7.4), 7.5 mmol/L EDTA, 0.15 mol/L NaCl at 37 °C. After 2 min incubation, 20 µL of Tris-HCl buffer containing 1.5 mmol/L chromogenic substrate S-2238 for thrombin or S-2765 for factor Xa was added, and the residual activity of thrombin or factor Xa was determined by measuring the change in the absorbance at 405 nm. The change rate of absorbance was proportional to the thrombin or factor Xa activity remaining in the incubation. No inhibition occurred in control experiments in which thrombin or factor Xa was incubated with HC-II or AT-III in the absence of the sulfated polysaccharide. Heparin was used as a positive control, and saline (0.9% NaCl) was used as a control.

### 3.12. Thrombolytic Activity In Vitro

The assay was carried out according to the method by Omura et al. [50]. The blood of Sprague–Dawley rats was drawn from the abdominal aorta and kept at room temperature until the big blood clot was completely formed. Subsequently, the clot was cut into appropriate pieces, weighed, and put into polyethylene tubes, respectively. The tubes were randomly divided into five experimental groups (10 clots/group): OCSH4 (3.75, 7.5, 15 mg/mL) group, saline group, and urokinase (100 U/mL) group. A sample (1 mL) was put into each tube, followed by shaking at 37 °C overnight. The residual clot was taken out from the tube. The liquid in the surface of the clot was removed. The wet weight of the residual clot was immediately determined. The lysis of the clot was calculated based on the equation: clot lytic rate (%) = (1 − W_b_/W_a_) × 100, W_a_ represents wet weight of the whole clot; W_b_ represents the wet weight of the residual clot.

### 3.13. Statistical Analysis

Bioassay results were analyzed using the statistical software GraphPad Prism 7.00 (La Jolla, CA, USA). The data were presented as means ± standard deviations (SD). Turkey’s test was used to compare the results and *p* < 0.05 was considered significant.

## 4. Conclusions

The sulfated polysaccharide OCSH4 from the green alga *C. oligoclada* was constituted by →6)-*β*-d-Gal*p*-(1→, *β*-d-Gal*p*-(1→, →6)-*α*-d-Glc*p*-(1→ and →3)-*β*-d-Gal*p*-(1→ units with sulfate esters at C-2/C-4 of →6)-*β*-d-Gal*p*-(1→, C-6 of →3)-*β*-d-Gal*p*-(1→ and C-3 of →6)-*α*-d-Glc*p*-(1→ units. The branches consisting of *β*-d-Gal*p*-(1→ and →6)-*β*-d-Gal*p*-(1→ units were at C-3 of →6)-*β*-d-Gal*p*-(1→ units. OCSH4 exhibited potent anticoagulant activities both in vitro and in vivo. OCSH4 largely inhibited the intrinsic coagulation factors VIII, IX, XI, and XII, and it inhibited the common coagulation factors II and V. Moreover, OCSH4 had a strong inhibitory effect on thrombin mediated by HC-II or AT-III, whereas its high inhibition of factor Xa was in an AT-III-dependent manner. The strong thrombolytic activity in vitro of OCSH4 was also revealed. Therefore, OCSH4 has potential to be developed into a novel anticoagulant agent for prevention and therapy of thrombotic diseases. There should be further studies on the antithrombotic activity, in vivo, of OCSH4, due to its prospects in drug development.

## Figures and Tables

**Table 1:** Results of methylation analyses of OCSH4 and dsOCSH4.

Methylated Alditol Acetate	Molar Percent Ratio		Linkage Pattern
	OCSH4	dsOCSH4	
1,5-Di-*O*-acetyl-2,3,4,6-tetra-*O*-methyl-galactitol	10.91	11.06	Gal*p*-(1→
1,3,5-Tri-*O*-acetyl-2,4,6-tri-*O*-methyl-galactitol	--	13.25	→3)-Gal*p*-(1→
1,5,6-Tri-*O*-acetyl-2,3,4-tri-*O*-methyl-glucitol	--	10.31	→6)-Glc*p*-(1→
1,5,6-Tri-*O*-acetyl-2,3,4-tri-*O*-methyl-galactitol	37.03	52.32	→6)-Gal*p*-(1→
1,4,5,6-Tetra-*O*-acetyl-2,3-di-*O*-methyl-galactitol	10.44	--	→4,6)-Gal*p*-(1→
1,3,5,6-Tetra-*O*-acetyl-2,4-di-*O*-methyl-glucitol	10.33	--	→3,6)-Glc*p*-(1→
1,3,5,6-Tetra-*O*-acetyl-2,4-di-*O*-methyl-galactitol	26.26	13.06	→3,6)-Gal*p*-(1→
1,2,5,6-Tetra-*O*-acetyl-3,4-di-*O*-methyl-galactitol	5.03	--	→2,6)-Gal*p*-(1→

**Table 2:** Signal assignments of NMR spectra of dsOCSH4.

Sugar Residues	Chemical Shifts (ppm)					
	H1/C1	H2/C2	H3/C3	H4/C4	H5/C5	H6/C6
A→6)-*α*-d-Glc*p*-(1→	5.01/99.43	3.86/71.10	3.80/73.59	3.73/70.48	3.71/71.63	4.00;4.09/69.59
B→3)-*β*-d-Gal*p*-(1→	4.73/104.99	3.80/71.19	3.96/75.63	4.08/70.30	3.73/73.59	3.80;3.86/62.05
C *β*-d-Gal*p*-(1→	4.61/104.09	3.71/71.72	3.69/71.89	4.00/70.39	3.96/73.59	3.86/62.14
D→3,6)-*β*-d-Gal*p*-(1→	4.53/104.99	3.56/71.01	3.74/76.06	3.72/71.01	3.80/73.68	3.96/69.68
E→6)-*β*-d-Gal*p*-(1→	4.51/104.42	3.73/71.89	3.58/71.63	3.99/70.58	3.97/73.50	3.96/69.68

Spectra were performed on an Agilent DD2 500M NMR spectrometer. Chemical shifts are referenced to internal acetone at 2.225 ppm for ^1^H and 31.07 ppm for ^13^C. Glc*p*: glucopyranose, Gal*p*: galactopyranose.

**Table 3:** Signal assignments of NMR spectra of OCSH4.

Sugar Residues	Chemical Shifts (ppm)					
	H1/C1	H2/C2	H3/C3	H4/C4	H5/C5	H6/C6
A→6)-*α*-d-Glc*p*(3SO_4_)-(1→	5.09/99.57	3.99/70.31	4.48/79.55	3.74/72.26	3.72/72.31	4.94;4.14/70.31
B→6)-*β*-d-Gal*p*(2SO_4_)-(1→	4.76/103.03	4.40/79.64	3.69/70.48	3.99/70.31	3.95/74.03	3.94/70.40
C *β*-d-Gal*p*-(1→	4.62/104.30	3.72/73.95	3.69/71.32	3.98/70.40	3.96/74.63	3.80/62.43
D→3)-*β*-d-Gal*p*(6SO_4_)-(1→	4.60/104.30	3.69/72.26	3.73/77.34	4.05/70.31	3.57/72.26	4.23/72.26
E→6)-*β*-d-Gal*p*(4SO_4_)-(1→	4.59/104.30	3.57/73.87	3.59/72.26	4.43/77.34	3.99/74.03	3.94/70.23
F→3,6)-*β*-d-Gal*p*-(1→	4.53/104.88	3.56/72.18	3.73/77.34	3.57/71.32	3.59/73.95	3.95/70.23
G→6)-*β*-d-Gal*p*-(1→	4.50/104.88	3.72/72.26	3.57/72.26	3.99/70.31	3.95/74.03	3.94/70.40

Spectra were performed on an Agilent DD2 500M NMR spectrometer. Chemical shifts are referenced to internal acetone at 2.225 ppm for ^1^H and 31.07 ppm for ^13^C. Glc*p*: glucopyranose, Gal*p*: galactopyranose.

**Table 4:** Result of anticoagulant activity assay of OCSH4 in vivo.

Samples	Concentration (mg/kg)	Clotting Time ^a^ (s)		
		APTT	TT	PT
OCSH4	0	18.2 ± 1.7	20.3 ± 0.9	15.3 ± 0.1
	1	39.7 ± 3.3 **	35.5 ± 2.6 **	15.2 ± 0.7
	3.75	88.9 ± 2.7 **	42.1 ± 1.2 **	15.6 ± 0.4
	7.5	129.6 ± 4.5 **^##^	60.4 ± 2.7 **	15.7 ± 0.9
	15	>200 **^##^	79.7 ± 3.4 **^#^	15.7 ± 1.1
Heparin	0	18.2 ± 1.7	20.3 ± 0.9	15.3 ± 0.1
	1	106.4 ± 3.7 **	60.2 ± 0.8 **	55.3 ± 1.7

^a^ Values were mean ± SD (*n* = 8). Significance: ** *p* < 0.01 vs. the control group; ^#^ *p* < 0.05, ^##^ *p* < 0.01 vs. the heparin group.

**Table 5:** Inhibition of coagulation factors, II, V, X, VIII, IX, XI and XII by OCSH4.

Coagulation Factors	Activities of Coagulation Factors (%) ^a^								
	OCSH4 (μg/mL)					Heparin (μg/mL)			
	0	5	10	25	50	0	0.1	1	5
II	101.8 ± 1.9	97.2 ± 2.9	98.4 ± 1.2	87.2 ± 1.2	63.7 ± 2.0	100.0 ± 0.9	90.7 ± 2.6	86.4 ± 2.1	75.2 ± 1.1
V	101.2 ± 2.21	98.7 ± 3.4	69.6 ± 1.8	35.5 ± 4.0	18.3 ± 1.1	99.9 ± 3.2	69.3 ± 4.7	41.6 ± 2.9	<1.0
VII	100.0 ± 2.1	64.7 ± 2.2	41.4 ± 1.7	6.8 ± 4.0	<1.0	100.4 ± 1.8	50.2 ± 2.7	1.4 ± 3.3	<1.0
IX	100.0 ± 1.0	79.0 ± 3.2	53.5 ± 0.7	12.1 ± 0.9	<1.0	100.9 ± 0.3	55.9 ± 0.7	2.3 ± 1.6	<1.0
X	99.8 ± 1.1	97.1 ± 0.8	95.2 ± 1.5	94.4 ± 2.0	94.2 ± 2.6	100.5 ± 1.3	97.8 ± 3.1	95.2 ± 0.5	82.1 ± 3.3
XI	100.0 ± 0.2	72.5 ± 1.4	47.7 ± 0.4	9.1 ± 1.2	<1.0	100.7 ± 1.2	42.0 ± 1.9	9.2 ± 1.1	<1.0
XII	102.0 ± 2.4	41.3 ± 1.2	27.0 ± 0.3	7.9 ± 1.6	<1.0	101.2 ± 1.9	59.7 ± 0.5	1.3 ± 0.2	<1.0

^a^ Values were mean ± SD (*n* = 3). The activities of coagulation factors were estimated by reference to calibration curves made by serial dilutions of standard human plasma. The standard human plasma with a dilution ratio of 1:5 (plasma/imidazole buffer, *v/v*) was used as a control.

## Supplementary Materials

The following are available online at https://www.mdpi.com/article/10.3390/md19100554/s1, Figure S1: IR spectrum, HPGPC, and HPLC chromatograms of dsOCSH4, Figure S2: NMR spectra of OCSH4.
