# Supplementary material for: Chemical Structure and Anticoagulant Property of a Novel Sulfated Polysaccharide from the Green Alga Cladophora oligoclada
Source: Mar Drugs. 2021 Sep 29;19(10):554. doi: 10.3390/md19100554 (PMC8540071; doi:10.3390/md19100554)
Supplement: Supplementary file 1 [file marinedrugs-19-00554-s001.zip › marinedrugs-1375251-supplementary.pdf]

SUPPLEMENTARY FIGURES

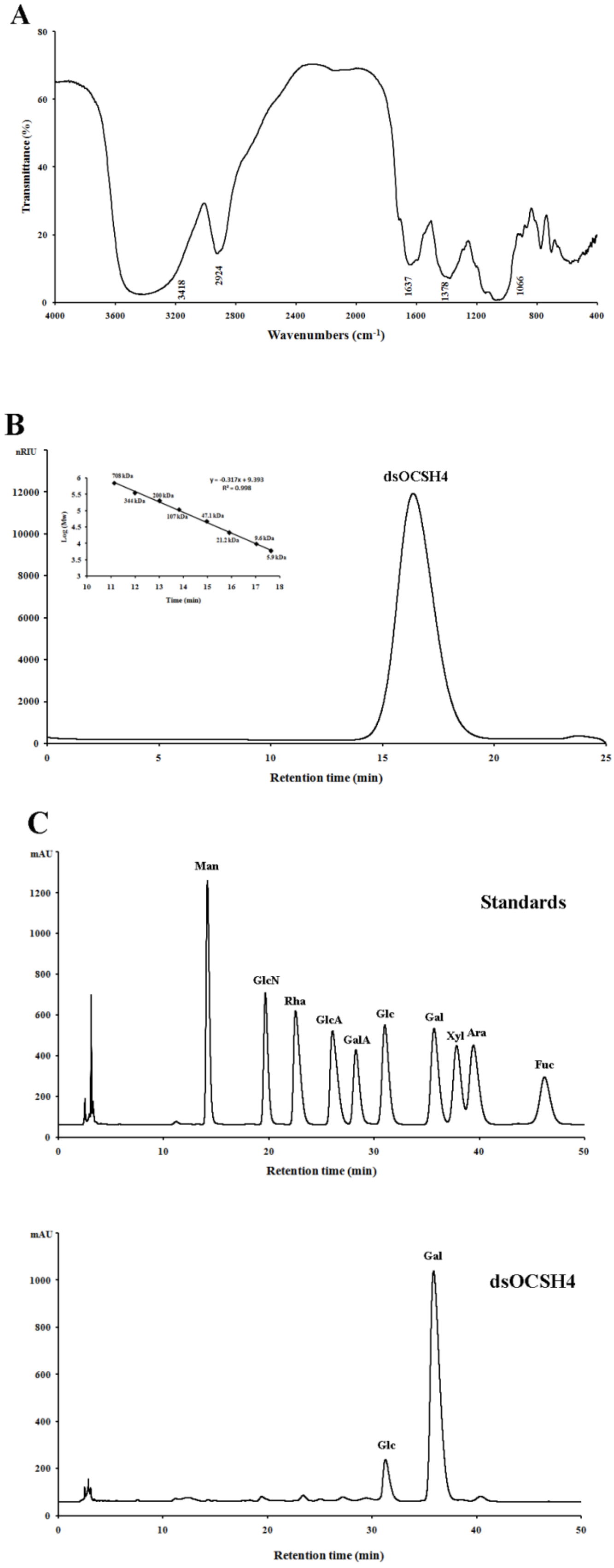

Figure S1. IR spectrum, HPGPC and HPLC chromatograms of dsOCSH4. **(A)** IR spectrum of dsOCSH4; **(B)** HPGPC chromatogram of dsOCSH4 on a Shodex OHpak SB-804 HQ

column and the standard curve of molecular weight; and **(C)** HPLC chromatogram for monosaccharide composition analysis of dsOCSH4 (Man: D-mannose, GlcN: D-glucosamine, Rha: L-rhamnose, GlcA: D-glucuronic acid, GalA: D-galacturonic acid, Glc: D-glucose, Gal: D-galactose, Xyl: D-xylose, Ara: L-arabinose, Fuc: L-fucose).

^{1}H NMR

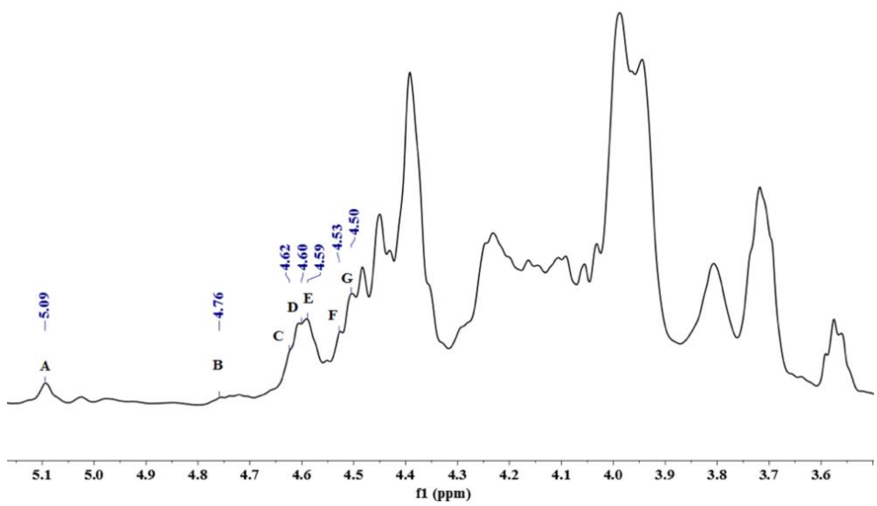

^{13}C NMR

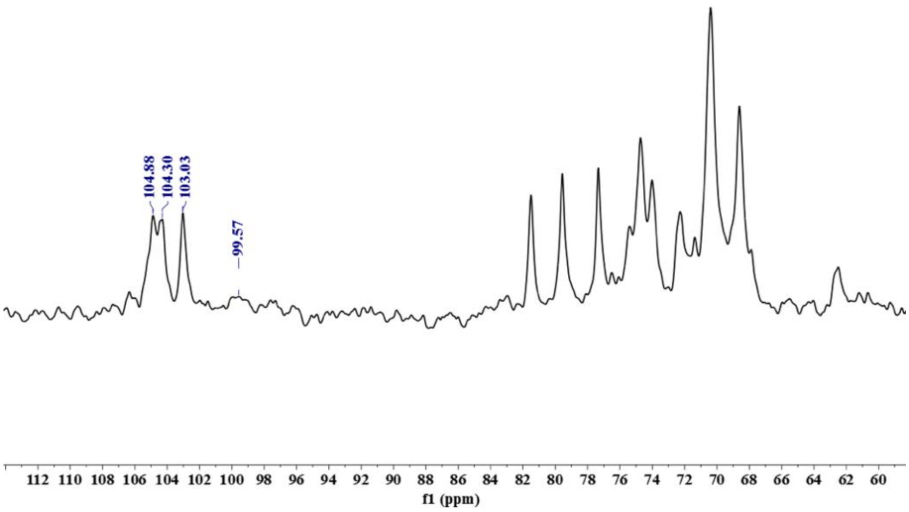

^{1}H–^{1}H COSY

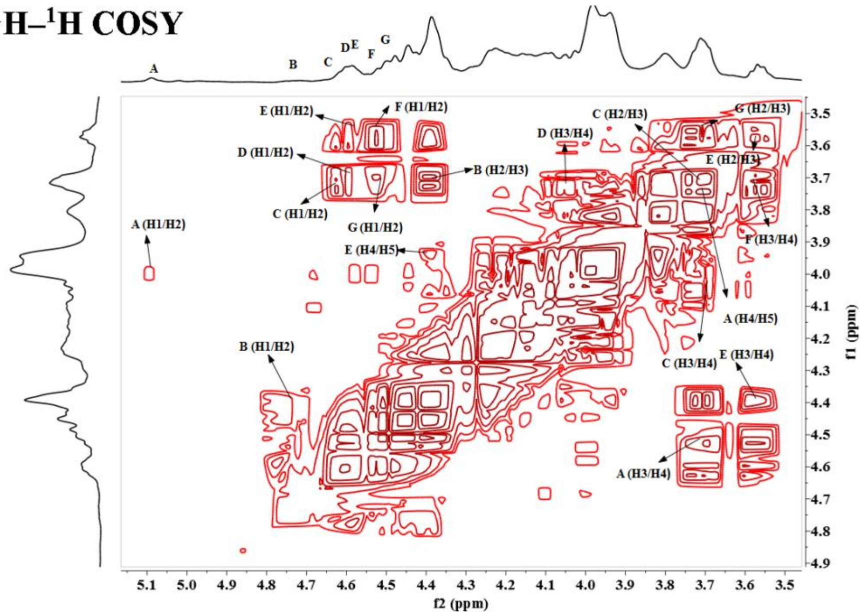

^{1}H–^{13}C HSQC

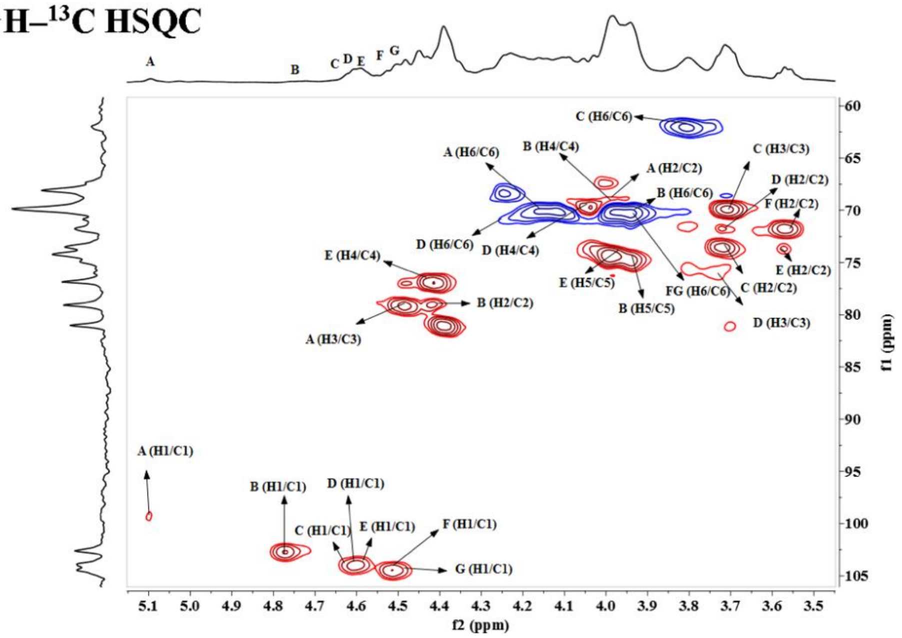

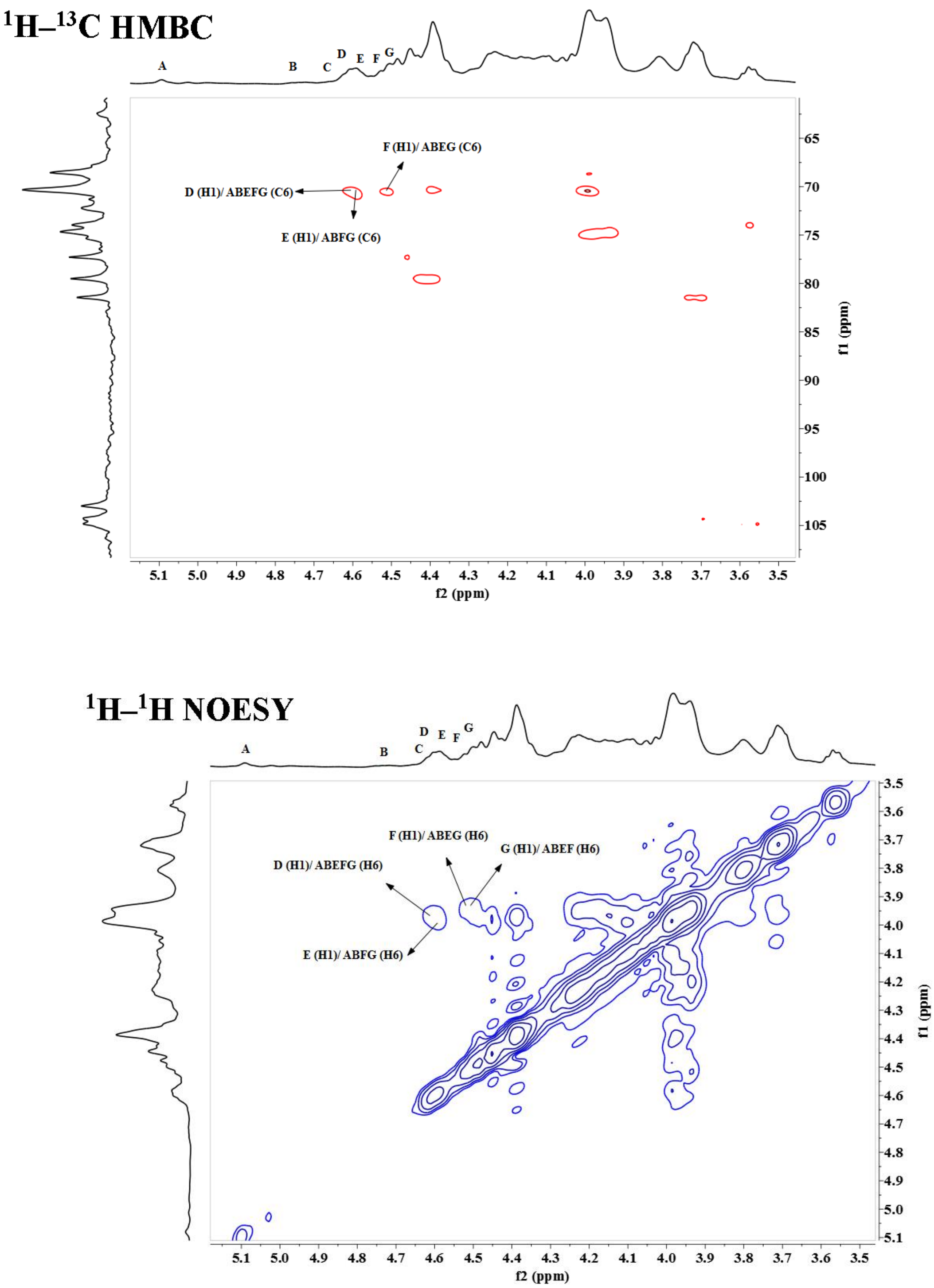

Figure S2. NMR spectra of OCSH4. Spectra were performed on an Agilent DD2 500M NMR spectrometer. Chemical shifts are referenced to internal acetone at 2.225 ppm for ^{1}H and 31.07 ppm for ^{13}C. A: →6)-*α*-D-Glc*p*(3SO$_4$)-(1→; B: →6)-β-D-Gal*p*(2SO$_4$)-(1→; C: β-D-Gal*p*-(1→; D: →3)-β-D-Gal*p*(6SO$_4$)-(1→; E: →6)-β-D-Gal*p*(4SO$_4$)-(1→; F: →3,6)-β-D-Gal*p*-(1→; G: →6)-β-D-Gal*p*-(1→. Glc*p*: glucopyranose, Gal*p*: galactopyran
